# T202. HUMOR-SKILLS TRAINING IN PATIENTS WITH SCHIZOPHRENIA: EFFECTS ON SYMPTOMS AND SOCIAL FUNCTIONING

**DOI:** 10.1093/schbul/sby016.478

**Published:** 2018-04-01

**Authors:** Irina Falkenberg, Florian Bitsch, Philipp Berger, Arne Nagels, Benjamin Straube

**Affiliations:** Philipps-University

## Abstract

**Background:**

Humor can provide a method of coping with a variety of stressful situations. Training of humor-related skills has proven effective in clinical samples, although humor training in patients with schizophrenia is relatively rare.

**Methods:**

In the present study, patients with schizophrenia have been randomly assigned to either a training of humor abilities or a training of social skills. Training effects on measures of psychopathology, psychosocial functioning and stress were compared between groups.

**Results:**

Preliminary analyses revealed that level of negative symptoms, stress and psychosocial dysfunction were significantly reduced in the humor group over the course of the training.

**Discussion:**

These results suggest that humor training may improve important clinical and functional outcomes in patients with schizophrenia.

